# Role of cathepsin S In periodontal wound healing–an in vitro study on human PDL cells

**DOI:** 10.1186/s12903-018-0518-2

**Published:** 2018-04-05

**Authors:** Svenja Memmert, Marjan Nokhbehsaim, Anna Damanaki, Andressa V. B. Nogueira, Alexandra K. Papadopoulou, Christina Piperi, Efthimia K. Basdra, Birgit Rath-Deschner, Werner Götz, Joni A. Cirelli, Andreas Jäger, James Deschner

**Affiliations:** 1 Section of Experimental Dento-Maxillo-Facial Medicine, Center of Dento-Maxillo-Facial Medicine, University of Bonn, Bonn, Germany; 2 Department of Orthodontics, Center of Dento-Maxillo-Facial Medicine, University of Bonn, Bonn, Germany; 3 Department of Diagnosis and Surgery, School of Dentistry at Araraquara, Sao Paulo State University, UNESP, Araraquara, Brazil; 4 Discipline of Orthodontics, Faculty of Dentistry, University of Sydney, Sydney, Australia; 5 Department of Biological Chemistry, Medical School, National and Kapodistrian University of Athens, Athens, Greece; 6 Noel Martin Visiting Chair, Faculty of Dentistry, University of Sydney, Sydney, Australia

**Keywords:** cathepsin S, periodontal ligament cells, wound closure, migration

## Abstract

**Background:**

Cathepsin S is a cysteine protease, which is expressed in human periodontal ligament (PDL) cells under inflammatory and infectious conditions. This in vitro study was established to investigate the effect of cathepsin S on PDL cell wound closure.

**Methods:**

An in vitro wound healing assay was used to monitor wound closure in wounded PDL cell monolayers for 72 h in the presence and absence of cathepsin S. In addition, the effects of cathepsin S on specific markers for apoptosis and proliferation were studied at transcriptional level. Changes in the proliferation rate due to cathepsin S stimulation were analyzed by an XTT assay, and the actions of cathepsin S on cell migration were investigated via live cell tracking. Additionally, PDL cell monolayers were treated with a toll-like receptor 2 agonist in the presence and absence of a cathepsin inhibitor to examine if periodontal bacteria can alter wound closure via cathepsins.

**Results:**

Cathepsin S enhanced significantly the in vitro wound healing rate by inducing proliferation and by increasing the speed of cell migration, but had no effect on apoptosis. Moreover, the toll-like receptor 2 agonist enhanced significantly the wound closure and this stimulatory effect was dependent on cathepsins.

**Conclusions:**

Our findings provide original evidence that cathepsin S stimulates PDL cell proliferation and migration and, thereby, wound closure, suggesting that this cysteine protease might play a critical role in periodontal remodeling and healing. In addition, cathepsins might be exploited by periodontal bacteria to regulate critical PDL cell functions.

## Background

Periodontitis is a highly-prevalent chronic inflammatory disease characterized by bone, attachment and even tooth loss [1]. Oral periodontopathogens are a prerequisite for the disease but not sufficient to induce periodontitis. Additional risk factors, such as smoking, genetic predisposition and certain systemic diseases contribute to the initiation and progression of periodontitis [2]. The periodontal microorganisms such as *Fusobacterium nucleatum* induce an inflammatory host response through binding to special receptors such as toll-like receptor (TLR) 2, which can ultimately result in the destruction of periodontal structures [3].

Periodontal ligament (PDL) cells are resident cells of the periodontium and have a critical role in tissue homeostasis, destruction and regeneration by their ability to synthesize and degrade collagen and other matrix molecules [4]. However, these cells can also participate in the immunoinflammatory processes of periodontitis [5]. Periodontal healing is determined by the type of cells that repopulate the root. By the application of regenerative treatment methods, which promote PDL cell proliferation, migration and attachment, the re-establishment of the initial periodontal tissue architecture is possible [6]. However, the outcomes of currently available regenerative treatment approaches are sometimes compromised by a number of factors and are not predictable [7, 8]. Therefore, the search for new molecules with a regenerative potential are a major goal in periodontology [9].

Cathepsin S (CTSS) is a lysosomal cysteine protease and has the ability to remain stable and active under neutral pH [10–12]. Therefore, it can evoke both intra- and extracellular activities. Intracellularly, CTSS functions as a processing enzyme and is critical for protein trafficking and secretion, while extracellularly it has a pivotal role in tissue remodeling [11]. This protease has the capacity to degrade multiple components of the extracellular matrix, such as collagen, elastin, fibronectin, laminin and proteolglycans [11, 13, 14]. Moreover, substrates of CTSS not only comprise antigenic as well as antimicrobial peptides but also play a fundamental role in antigen processing and presentation [11, 15, 16]. Additionally, it has been shown that CTSS promotes cell migration [17]. Hence, these functions of CTSS suggest a complex role in immunoinflammatory diseases and healing processes [14, 18, 19].

CTSS is not produced ubiquitously and its synthesis seemed to be restricted to immunocompetent cells, such as macrophages, lymphocytes and dendritic cells [14, 19]. Previously, we have found that CTSS is also secreted by PDL cells and that its synthesis is regulated by inflammatory and microbial stimuli, suggesting strongly a role of this protease in oral inflammatory diseases [20]. Moreover, in gingival biopsies from sites of periodontitis, CTSS was identified as a hub protein in the protein-protein interaction network of differentially expressed genes, also suggesting an involvement of CTSS in periodontitis [21]. Therefore, the aim of this in vitro study was to investigate the effects of CTSS on PDL cell wound closure.

## Methods

### Isolation and characterization of PDL cells

Written informed consent and approval of the Ethics Committee of the University of Bonn were obtained (#117/15). Human PDL cells were taken from caries-free and periodontally healthy teeth of 5 donors (mean age: 14.6 years, min/max: 13/19 years; 3 males/2 females), who had to undergo tooth extractions for orthodontic reasons [22, 23]. Cells were harvested from the medial part of the tooth root and grown in Dulbecco’s minimal essential medium (DMEM, Invitrogen, Karlsruhe, Germany) supplemented with 10% fetal bovine serum (FBS, Invitrogen), 100 units penicillin and 100 μg/mL streptomycin (Invitrogen) in a humidified atmosphere of 5% CO_2_ at 37 °C. Cells between passages 3 to 5 were phenotyped according to Basdra and Komposch and used for experiments at 60%-100% confluence, depending on the individual protocol of each experiment [22].

### Cell treatment

One day prior to the experiments, the FBS concentration was reduced to 1%. PDL cells were incubated with the active form of CTSS (1 ng/μl; activity 143.4 U/mL; Calbiochem, San Diego, CA, USA) for up to 72 h [24]. In a subset of experiments, PDL cells were incubated with a TLR2 agonist (1 μg/ml; Pam3CSK4; Invivogen, San Diego, CA, USA) in the presence and absence of a cathepsin inhibitor (50 μM; Z-FA-FMK; Santa Cruz Biotechnology, Dallas, TX, USA) for 24 h [25, 26].

### In vitro wound healing assay

To analyze the in vitro wound healing, a well-established in vitro wound healing model was applied as in our previous studies [27–29]. Briefly, PDL cells were cultured on 35 mm plastic culture dishes and grown to 100% confluence. One day after reduction of FBS concentration, a 3–4 mm-wide wound was inflicted in a standardized manner so that cell free areas were created in the cell monolayers. Afterwards, all non-adherent cells were removed through multiple washing steps with DMEM. The wounded monolayers were then cultured in the presence and absence of CTSS for 72 h or the TLR2 agonist in combination with or without the cathepsin inhibitor for 24 h, as described above. By using a JuLI™ Br and the JuLI™ Br PC software (both NanoEnTek, Seoul, Korea), the wound closure was monitored over time and, subsequently, the wound healing rates were calculated.

### Cell migration

Live cell imaging was applied to monitor cell migration over a period of 24 h. Cells were grown to 100% confluence, and cell free areas were created in a standardized manner. Subsequently, cells were treated as described above. The live cell tracker JuLI™ Br device and the JuLI™ Br PC software were used to capture images and to monitor the migration of individual cells. In each group and donor, 6 cells which had moved the farthest into the cell-free area after 24 h, were marked and traced. Images were transferred to and analyzed by the freely available image-processing software Image J 1.43 [30].

### Gene expression

PDL cell monolayers were cultured in the presence and absence of CTSS for 24 h. Afterwards, RNA extraction was performed with a commercially available RNA extraction kit (RNeasy Protect Minikit, Qiagen, Hilden, Germany) and 1 μg of RNA was converted into cDNA by reverse-transcription with the IScript Select cDNA Synthesis Kit (Bio-Rad Laboratories, Munich, Germany). Expressions of Proliferating Cell Nuclear Antigen (PCNA) and p53, which are markers for cell proliferation and cell death, respectively, were determined by real-time PCR by using the iCyler iQ detection system (Bio-Rad Laboratories) [31, 32]. cDNA expression of 1 μl of cDNA was detected via real-time PCR in a 25 μl reaction mixture containing 2.5 μl respective QuantiTect Primer assay (Qiagen), 12.5 μl QuantiTect SYBR Green Master Mix (Qiagen) and 9 μl of nuclease free water, as recommended by the manufacturer. The protocol comprised a heating phase at 95 °C for 5 min to activate the enzyme, followed by 40 cycles of a denaturation step at 95 °C for 10 s and a combined annealing/extension step at 60 °C for 30 s. Melting point analysis was performed after each run. For normalization, the housekeeping gene GAPDH was used.

### Cell proliferation

Cell proliferation was analyzed by using the AppliChem Cell Proliferation Kit XTT (AppliChem, Darmstadt, Germany). In a 96-well-plate, PDL cells (5.000/well) were grown to 60% confluence and stimulated with CTSS for up to 24 h. XTT reaction solution was added to the medium 4 h before measurements. Finally, the absorbance was determined with a microplate reader (PowerWave x, BioTek Instruments, Winooski, VT, USA) at 475 nm.

### Statistical Analysis

The IBM SPSS Statistics software (Version 22, IBM SPSS, Chicago, IL, USA) was used for statistical analysis. Mean values and standard errors of the mean (SEM) were calculated for quantitative data. All experiments were performed in triplicate and repeated at least twice. For statistical comparison of the groups, the t- and Mann-Whitney-U tests were applied. Differences between groups were considered significant at *p* < 0.05.

## Results

### Effect of CTSS on wound healing

**Fig. 1:**
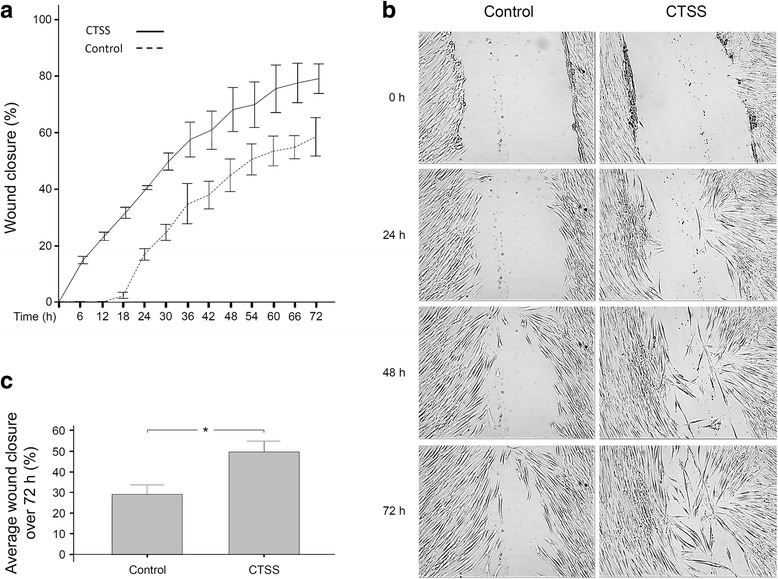
**a** Wound closure of PDL cell monolayers in the presence or absence of CTSS (1 ng/μl) over 72 h. The wound closure, i.e., the percentage of cell coverage of the initially cell-free zones created by wounding, were analyzed by live cell imaging. Mean ± SEM. **b** Wound closure of PDL cell monolayers in the presence or absence of CTSS (1 ng/μl) at 0 h, 24 h, 48 h and 72 h. Images from one representative donor are shown. **c** Average wound closure of PDL cell monolayers shown in **a**. Mean ± SEM (*n* = 26), * significant (*p* < 0.05) difference between groups

Since we recently found that PDL cells produce CTSS and other studies have demonstrated that CTSS affects cell proliferation and migration, we analyzed CTSS effects on wound closure of PDL cell monolayers [17, 18, 20]. After incubation of wounded PDL cell monolayers with CTSS, the wound closure was followed up over 72 h. Wounded monolayers without CTSS treatment served as control. As shown in Fig. 1a and b, wound closure started earlier in the CTSS-treated group as compared to control. Moreover, the wound fill rate was higher in the CTSS-treatment group at all time points. At 72 h, the wound closure was 79% in the treatment group and 58% in control. When the average wound closure over 72 h was calculated, the wound fill rate in the CTSS-treated group and control were 49% and 29%, respectively, demonstrating a significant difference between both groups (Fig. 1c).

### Influence of CTSS on migration

**Fig. 2:**
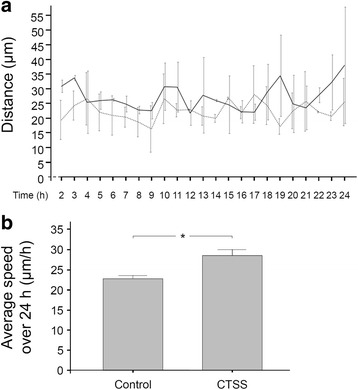
**a** PDL cell migration, i.e., the distance which the cells covered within 1 h, in the presence or absence of CTSS (1 ng/μl) over 24 h. Mean ± SEM (*n* = 12). **b** Average speed of PDL cells shown in **a**. Mean ± SEM, * significant (*p* < 0.05) difference between groups

The wound fill rate is also determined by the speed of cell migration. Therefore, we also studied the effects of CTSS on this cell function. In wounded PDL cell monolayers in the presence and absence of CTSS, the cell migration was monitored by live cell imaging over a period of 24 h. Cells which had moved the farthest into the cell-free area after 24 h, were marked and traced. As shown in Fig. 2a, CTSS treatment of cells resulted in an accelerated migration, as compared to control. For the majority of time points, the distance which the cells covered within 1 h, was greater in CTSS-treated groups than in control. When the average speed over 24 h was calculated, the migration was significantly faster in the CTSS group (27.16 μm/h) than in control (22.34 μm/h), as depicted in Fig. 2b.

### Actions of CTSS on proliferation and apoptosis

**Fig. 3:**
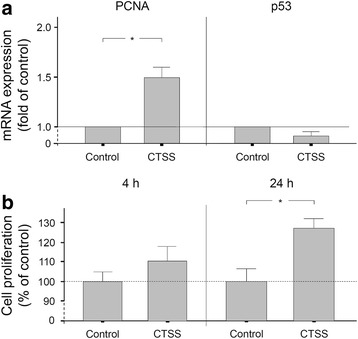
**a** PCNA and p53 gene expressions in the presence or absence of CTSS (1 ng/μl) at 24 h. Mean ± SEM (*n* = 9), * significant (*p* < 0.05) difference between groups. **b** PDL cell proliferation in the presence or absence of CTSS (1 ng/μl) at 4 h and 24 h. Mean ± SEM (*n* = 24), * significant (*p* < 0.05) difference between groups

Since wound closure also depends on the cell number, which is determined by proliferation and apoptosis, we also studied the CTSS actions on these cell functions. Treatment of PDL cells with CTSS resulted in a significantly increased expression of PCNA, a specific marker of cell proliferation (Fig. 3a). However, no difference between groups was observed with regard to p53 expression, a marker of apoptosis (Fig. 3a). To confirm our observation for PCNA at transcriptional level, we also studied the proliferation of PDL cells in the presence and absence of CTSS by using a commercially available XTT assay. Although there was a trend to an increased proliferation in the CTSS group, the difference did not reach significance at 4 h (Fig. 3b). However, incubation of PDL cells with CTSS for 24 h induced a significantly enhanced proliferation rate by 27% (Fig. 3b).

### Involvement of cathepsins in TLR2 effects on wound closure

**Fig. 4:**
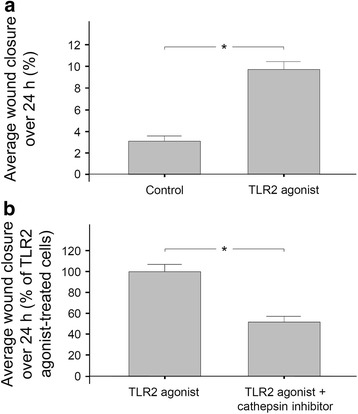
**a** Average wound closure of PDL cell monolayers in the presence or absence of a TLR2 agonist (Pam3CSK4; 1 μg/ml) over 24 h. Mean ± SEM. * significant (*p* < 0.05) difference between groups. **b** Average wound closure of PDL cell monolayers treated with a TLR2 agonist in the presence and absence of a cathepsin inhibitor (Z-FA-FMK; 50 μM) over 24 h. Mean ± SEM, * significant (*p* < 0.05) difference between groups

The aforementioned experiments demonstrated that CTSS accelerates wound closure, and our previous experiments had revealed that *F. nucleatum*, which is able to activate TLR2, upregulates CTSS [20]. Therefore, we next sought to examine whether a TLR2 agonist would enhance the wound closure and whether such a potential stimulatory effect would involve cathepsins. As shown in fig. 4a, incubation of PDL cell monolayers with a TLR2 agonist enhanced significantly the average wound closure by approximately 70% over 24 h. However, when the TLR2 agonist-treated monolayers were simultaneously exposed to a cathepsin inhibitor, the enhanced wound closure was significantly reduced by 50% (Fig. 4b).

## Discussion

The present study investigated the effects of CTSS on PDL cell wound closure. Our findings provide original evidence that CTSS stimulates PDL cell proliferation and migration and, thereby, wound closure, suggesting that this cysteine protease might play a critical role in periodontal remodeling and healing. Moreover, TLR2 activation also accelerates the PDL cell wound closure and this stimulatory effect depends on cathepsins, suggesting that cathepsins might be exploited by periodontal bacteria to regulate critical PDL cell functions.

CTSS has been broadly implicated in health and pathology including autoimmune diseases, allergic inflammation and asthma, diabetes and obesity, cardiovascular and pulmonary diseases as well as cancer [33]. CTSS is a lysosomal cysteine protease capable of degrading components of the extracellular matrix, such as collagen, elastin, fibronectin, laminin and proteoglycans, suggesting a pivotal role in tissue homeostasis and repair [11, 13, 14]. This assumption is further supported by the observation that CTSS promotes cell migration and, additionally, regulates osteoblast differentiation and bone remodeling [17, 18, 34]. The synthesis of CTSS by periodontal cells and its role in periodontal tissues has been neglected so far. Our previous experiments have provided novel evidence that PDL cells can produce this cysteine protease [20]. Periodontal therapy results in wounding of the periodontal tissues and, subsequently, wound healing is initiated. Ideally, the healing is characterized by the restoration of the original tissue structure, form and function. However, periodontal regeneration requires appropriate PDL cell proliferation and migration. Therefore, in the present study, we sought to investigate possible actions of CTSS on PDL cells with a special focus on proliferation, migration and wound healing in vitro. The present experiments revealed that these cell functions are regulated by CTSS. The cysteine protease caused a significant upregulation of PCNA, a specific marker of proliferation, and proliferation, as assessed by an XTT assay. Moreover, our experiments showed that CTSS also increased the speed of PDL cell migration. Since both proliferation and migration determine the would fill rate, our observation that CTSS promotes wound healing is supported by these findings.

Gonzales et al. studied the gene expression in gingival biopsies from Rhesus monkeys and advocated a role for CTSS in periodontitis [35]. Furthermore, CTSS was identified as a hub protein in the protein-protein interaction network of differentially expressed genes, also indicating an involvement of this protease in periodontal diseases [21]. The results of our previous study which showed an increased production of CTSS by periodontal cells in response to inflammatory and infectious stimuli concur very well with these reports [20]. However, the findings of the present study demonstrate that CTSS might not only play a role in periodontal tissue destruction but also healing.

Notably, exposure of PDL cell monolayers to the TLR2 agonist resulted in a significantly accelerated wound closure in our study. Further experiments revealed that the stimulation of the in vitro wound healing by the TLR2 agonist was dependent on cathepsins. Interestingly, inhibition of TLR2 has been shown to reduce the CTSS gene expression in human endothelial cells, supporting our data [36]. Our observation is of major importance, because it links the experiments of this study with our previous findings [20]. Moreover, since we used a TLR2 agonist and an unspecific cathepsin inhibitor, this result may have an even broader significance for the understanding of periodontal diseases, because periodontitis is not only caused by a single bacterium or mediated by a single member of the cathepsin family. Whether the increased wound closure in the presence of the TLR2 agonist, as observed in our experiments, might be an attempt of the cells to maintain tissue homeostasis, has to be clarified in further studies.

Cathepsin K (CTSK), another member of the cathepsin family, has also been associated with periodontal diseases [37–39]. Interestingly, CTSS has the ability to degrade CTSK, indicating complex interactions between both cathepsins [40]. Further research should also focus on the role of CTSK in PDL cell proliferation, migration and wound closure.

Although not investigated in our study, the stimulatory effects of CTSS on cell migration and proliferation might involve TLR2-mediated p38/Akt signaling activation and histone deacetylase 6, as it has been disclosed in vascular smooth muscle cells by Wu and colleagues [18]. Another study on macrophages suggests that CTSS promotes cell migration through degradation of elastic fiber integrity [17]. Furthermore, CTSS might influence proliferation of PDL cells via peroxisome proliferator-activated receptor-gamma, as revealed in human umbilical vein endothelial cells [41]. Further studies should focus on the mechanisms underlying the stimulatory effects of CTSS on wound healing in periodontal cells.

In the present study, CTSS was used at concentrations of 1 ng/μl, as used in our previous experiments and in studies by other investigators [24]. Furthermore, we applied an established vitro wound healing model which is commonly used [27–29, 42, 43]. Nonetheless, the limitation of any in vitro model, including this one, is that it cannot fully mimic the plethora of actions and interactions of different cell and tissue types that take place in vivo [44]. Our experiments focussed on PDL cells, which constitute a heterogenous cell population, because they are critical for both periodontal destruction and regeneration. By phenotyping the cells before our experiments, we confirmed their ability to differentiate into osteoblastic cells. But as no osteogenic medium was applied in the experiments, the cells acquired a fibroblastic phenotype. Future studies should also examine the actions of CTSS on other periodontal cells involved in periodontal homeostasis and repair. Moreover, in order to further explore the role of CTSS in a more complex environment, an experimental periodontitis model in CTSS knock-out mice might be helpful.

## Conclusions

In summary, our study investigated the actions of CTSS on PDL cell wound closure. Our findings provide original evidence that cathepsin S stimulates PDL cell proliferation and migration and, thereby, wound closure, suggesting that this cysteine protease might play a critical role in periodontal remodeling and healing. In addition, cathepsins might be exploited by periodontal bacteria to regulate critical PDL cell functions.

## Abbreviations

CTSK: Cathepsin K
CTSS: Cathepsin S
DMEM: Dulbecco’s minimal essential medium
FBS: Fetal bovine serum
PCNA: Proliferating cell nuclear antigen
PDL: Periodontal ligament
SEM: Standard errors of the mean
TLR: Toll-like receptor.
